# Central insulin‐like growth factor‐1 (IGF‐1) restores whole‐body insulin action in a model of age‐related insulin resistance and IGF‐1 decline

**DOI:** 10.1111/acel.12415

**Published:** 2015-11-04

**Authors:** Derek M. Huffman, Gabriela Farias Quipildor, Kai Mao, Xueying Zhang, Junxiang Wan, Pasha Apontes, Pinchas Cohen, Nir Barzilai

**Affiliations:** ^1^ Division of EndocrinologyDepartment of MedicineAlbert Einstein College of MedicineBronxNYUSA; ^2^ Department of Molecular PharmacologyAlbert Einstein College of MedicineBronxNYUSA; ^3^ Institute for Aging ResearchAlbert Einstein College of MedicineBronxNY10461USA; ^4^ Institute of ZoologyChinese Academy of Sciences1 Beichen West RoadChaoyangBeijing100101China; ^5^ Davis School of GerontologyUniversity of Southern CaliforniaLos AngelesCA90089USA; ^6^ Department of GeneticsAlbert Einstein College of MedicineBronxNY10461USA

**Keywords:** aging, central nervous system, endocrinology, glucose metabolism, insulin resistance, insulin‐like growth factor, animal models

## Abstract

Low insulin‐like growth factor‐1 (IGF‐1) signaling is associated with improved longevity, but is paradoxically linked with several age‐related diseases in humans. Insulin‐like growth factor‐1 has proven to be particularly beneficial to the brain, where it confers protection against features of neuronal and cognitive decline. While aging is characterized by central insulin resistance in the face of hyperinsulinemia, the somatotropic axis markedly declines in older humans. Thus, we hypothesized that increasing IGF‐1 in the brain may prove to be a novel therapeutic alternative to overcome central insulin resistance and restore whole‐body insulin action in aging. Utilizing hyperinsulinemic‐euglycemic clamps, we show that old insulin‐resistant rats with age‐related declines in IGF‐1 level demonstrate markedly improved whole‐body insulin action, when treated with central IGF‐1, as compared to central vehicle or insulin (*P *<* *0.05). Furthermore, central IGF‐1, but not insulin, suppressed hepatic glucose production and increased glucose disposal rates in aging rats (*P *<* *0.05). Taken together, IGF‐1 action in the brain and periphery provides a ‘balance’ between its beneficial and detrimental actions. Therefore, we propose that strategies aimed at ‘tipping the balance’ of IGF‐1 action centrally are the optimal approach to achieve healthy aging and longevity in humans.

## Introduction

Diminished growth hormone/insulin‐like growth factor‐1 (GH/IGF‐1) signaling improves longevity across nature, including mice heterozygous for the IGF‐1 receptor (IGF‐1R) (Holzenberger *et al*., 2003; Barzilai *et al*., 2012). Moreover, low IGF‐1 action is relevant to humans because of its link to less cancer risk (Renehan *et al*., 2004), an observation that spurred the development of IGF‐1R antagonists as a clinical cancer treatment (Pollak, 2012). Furthermore, our group has reported on a novel, functional mutation in the human *IGF‐1R* gene (*R407H*) that is enriched in centenarians and results in attenuated IGF‐1R signaling (Suh *et al*., 2008). Similarly, low IGF‐1 levels predict greater life expectancy in exceptionally long‐lived individuals (Milman *et al*., 2014), further supporting this pathway as relevant to human aging.

Paradoxically, low IGF‐1 levels are associated with increased risk for numerous diseases of aging in humans, including cardiovascular disease (CVD) (Juul *et al*., 2002), type 2 diabetes (T2DM) (Sandhu *et al*., 2002), and frailty (Leng *et al*., 2004). Moreover, it was recently shown that long‐lived *daf‐2* mutants spent a larger proportion of life in a frail state than wild‐type *Caenorhabditis elegans* (Bansal *et al*., 2015). Insulin‐like growth factor‐1 is also beneficial to the developing and aging central nervous system (CNS), including protection against cognitive and neurosensory deficits, depressive‐like symptoms, and neurodegeneration (Westwood *et al*., 2014). Further, while GH receptor (and IGF‐1)‐deficient individuals were protected against CVD and T2DM, they suffered from a higher rate of convulsive disorders (Guevara‐Aguirre *et al*., 2011), further highlighting the importance of this axis in the brain.

The notion that low IGF‐1 action may be protective from some diseases, such as cancer, but predispose to others, is particularly relevant to older humans, where somatopause leads to age‐related declines in GH/IGF‐1 (Ashpole *et al*., 2014). This prompted many investigators to suspect that the decline of this axis may be causal to many aging phenotypes and that replacement could reverse some manifestations of aging (Rudman *et al*., 1990). However, beneficial outcomes could not be validated in subsequent trials (Liu *et al*., 2007), which, coupled with numerous side effects linked to chronic treatment, have precluded aging as an indication for its clinical use (Liu *et al*., 2007).

Although strategies aimed at restoring systemic GH/IGF‐1 levels in older humans have not proven feasible, many benefits of IGF‐1 can be alternatively achieved by targeting its action centrally, rather than peripherally. Indeed, central IGF‐1 in rodents confers neuroprotection and rescue of age‐related deficits in neuronal and cognitive function (Piriz *et al*., 2011; Deak & Sonntag, 2012). Further, our group extended upon the known benefits of IGF‐1 in the brain, by showing that similar to insulin, central IGF‐1 could improve insulin sensitivity in young animals (Muzumdar *et al*., 2006a). As aging is characterized not only by declining IGF‐1 levels, but also by hypothalamic insulin (Garcia‐San Frutos *et al*., 2007) and leptin resistance (Scarpace *et al*., 2000), we reasoned that centrally targeting IGF‐1 may prove to be a viable alternative for restoring whole‐body insulin action in aging.

## Results

**Figure 1:**
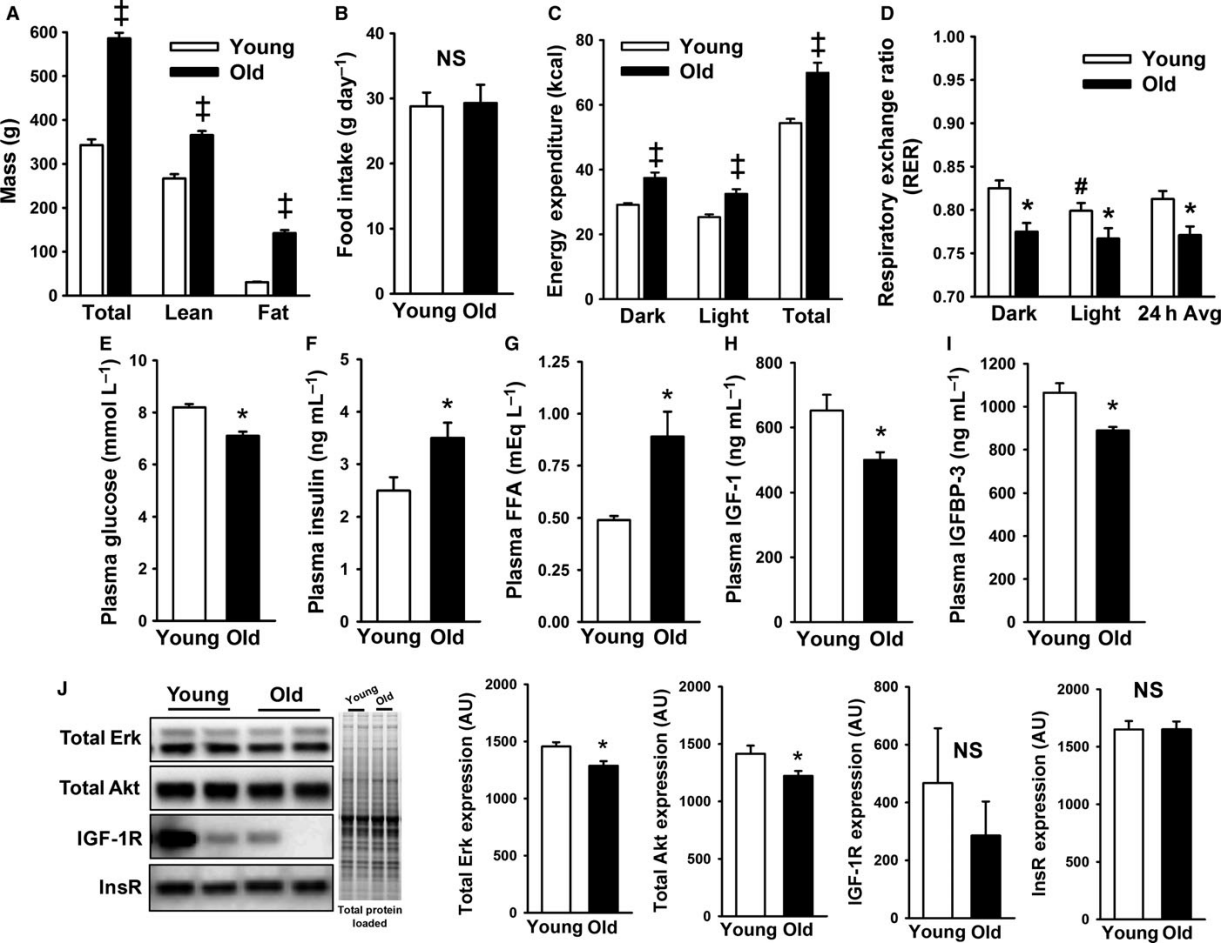
Phenotypic characteristics of young and old male Sprague–Dawley rats. (A) Old rats were heavier with more adiposity, (B) but food intake was similar between groups (*n *=* *8 per group). (C,D) Indirect calorimetry confirmed that old animals had greater energy expenditure and lower respiratory exchange ratio during the light and dark photoperiod (*n *=* *8 per group). (E–I) Plasma measures revealed several differences between young and old animals, including old animals having (E) lower glucose, but greater (F) insulin and (G) free fatty acids levels, while (H) insulin‐like growth factor‐1 (IGF‐1) and (I) IGFBP‐3 levels were reduced with aging (*n *=* *12 per group). (J) Western blots of mediobasal hypothalamus tissue detected a slight, but significant decrease in total Akt levels with aging, but no significant differences were observed for total Erk, IGF‐1R, or InsR levels between young and old (*n *=* *12 per group). *P <* *0.05, ^‡^ *P *<* *0.001, ^#^ *P *=* *0.06 vs. dark photoperiod.

To test our hypothesis, we studied young (3 month) and old (20 month) male Sprague–Dawley (SD) rats. We initially confirmed that old males were heavier and had greater lean and fat mass, compared to their younger counterparts (*P *<* *0.001; Fig. 1A). While food intake was similar (Fig. 1B), old animals had greater 24‐h energy expenditure (*P *<* *0.001; Fig. 1C), and a lower respiratory exchange ratio (RER), suggesting an impaired ability to use carbohydrate, relative to young rats (*P *<* *0.01; Fig. 1D). Interestingly, old SD rats had lower glucose levels (*P *<* *0.01; Fig. 1E), but were hyperinsulinemic (*P *<* *0.01; Fig. 1F) with greater plasma‐free fatty acids (FFA) (*P *<* *0.001; Fig. 1G), and lower IGF‐1 (*P *<* *0.05; Fig. 1H) and IGFBP‐3 levels (*P *<* *0.05; Fig. 1I). In the mediobasal hypothalamus (MBH), a slight but significant reduction in total Akt protein was observed with aging (*P *<* *0.05; Fig. 1J). However, IGF‐1R (*P *=* *0.47), as well as total Erk and insulin receptor (InsR) content, was unchanged in the aging MBH (Fig. 1J).

**Figure 2:**
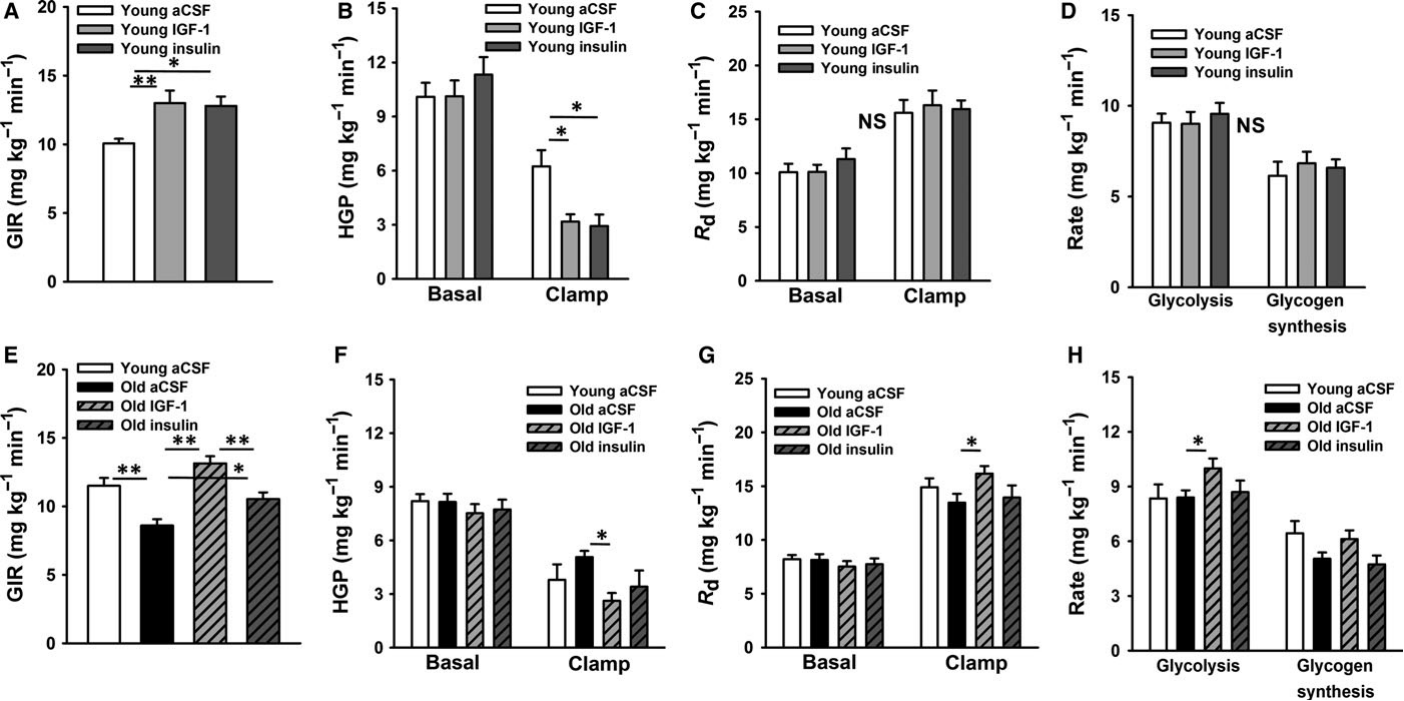
Results for the hyperinsulinemic‐euglycemic clamp study in young (A–D) and young vs. old rat experiments (E,F), respectively. (A) In young animals (*n *=* *5–9 per group), the glucose infusion rate (GIR) required to maintain euglycemia during a 3 mU kg^−1^ min^−1^ clamp was similarly increased by central insulin‐like growth factor‐1 (IGF‐1) (1 μg total dose) or insulin (30 μU total dose). (B) This was due to enhanced suppression of hepatic glucose production (HGP), (C) as glucose disposal, (D) whole‐body glycolysis and glycogen synthesis rates were not significantly altered. We next treated old animals with intracerebroventricular insulin or IGF‐1 (*n *=* *5–7 per group; E–H). (E) When compared to young controls, old animals had a significantly lower GIR, but central IGF‐1 markedly increased the GIR, as compared to artificial cerebral spinal fluid or insulin in old animals. (F) Central IGF‐1, but not central insulin, significantly suppressed HGP, as compared to old controls. (G) Likewise, central IGF‐1, but not central insulin, significantly increased glucose disposal rates (*R*_d_), as compared to old controls, (H) which was largely due to an increase in whole‐body glycolysis. *P <* *0.05, **P <* *0.01.

In order to determine the relative efficacy of central IGF‐1 vs. central insulin to improve insulin action in aging, we performed hyperinsulinemic‐euglycemic clamp studies with intracerebroventricular (ICV) infusions of artificial cerebral spinal fluid (aCSF), IGF‐1, or insulin, in male SD rats, as described (Muzumdar *et al*., 2006a). In an initial investigation, we studied young male animals (Fig. 2A–D; *n *=* *5–9 per group), in order to establish the ICV doses to be employed in the aging experiment (Fig. 2E–H). Utilizing ICV doses of insulin (30 μU) (Obici *et al*., 2002) or IGF‐1 (1 μg) (Muzumdar *et al*., 2006a), previously shown by our group to improve insulin sensitivity, we confirmed identical responses to these ligands on major readouts of glucose metabolism during the clamp in young rats. Specifically, we observed a similar increase in the glucose infusion rate (GIR) (*P *<* *0.05; Fig. 2A) and suppression of hepatic glucose production (HGP) (*P *<* *0.05; Fig. 2B), without alterations in glucose disposal (*R*_d_), whole‐body glycolysis, or glycogen synthesis rates (Fig. 2C,D).

Next, we investigated the ability of central insulin and IGF‐1 at the determined doses, to modulate glucose metabolism in aging. Four groups of young and aged male SD rats were studied (*n *=* *5–7 per group), including young controls (aCSF vehicle), old controls (aCSF vehicle), old IGF‐1, and old insulin. Clamp study results for these groups are shown in Fig. 2E–H. Under basal conditions, no differences were observed among groups for HGP or *R*_d_. However, the GIR required to maintain euglycemia under hyperinsulinemic conditions (3 mU kg^−1^ min^−1^ clamp) was lower in old aCSF controls, as compared to young aCSF (*P *<* *0.05; Fig. 2E). Remarkably, central IGF‐1 profoundly increased the GIR in old rats by ~ 53% over old controls (*P *<* *0.05; Fig. 2E), to levels numerically exceeding the GIR observed in young controls (*P *=* *0.08). In contrast, central insulin significantly increased the GIR by ~ 22% over old controls (*P *<* *0.05), but this more modest response was significantly less than achieved with central IGF‐1 administration (*P *<* *0.05; Fig. 2E). Furthermore, a separate dose response study with insulin confirmed a similar increase in the GIR by 30 μU or 22 mU ICV insulin (equimolar to IGF‐1) in old animals (not shown), suggesting that the less robust response to insulin was not dose dependent.

The ability of central IGF‐1 to restore whole‐body insulin action in old animals was attributed to both a suppression of HGP (*P *<* *0.05; Fig. 2F) and an increase in *R*_d_ (*P *<* *0.05; Fig. 2G), which was accompanied by a significant increase in whole‐body glycolysis (*P *<* *0.05; Fig. 2H). However, central insulin failed to significantly suppress HGP (*P *=* *0.12; Fig. 2F) or increase glucose uptake (*P *=* *0.97; Fig. 2G) in old animals. These observations were not attributable to differences in blood glucose or insulin levels during the clamp, as they were confirmed to be similar among groups (data not shown).

## Discussion

Here, we tested the hypothesis that central IGF‐1 is uniquely capable of restoring whole‐body insulin action in a model of age‐related insulin resistance and declining IGF‐1 levels. Indeed, while central insulin was minimally effective at improving whole‐body insulin action in old animals, central IGF‐1 acted via a dual mechanism, involving suppression of HGP and increased glucose disposal, to markedly improve insulin sensitivity. This observation, coupled with the known protective effects of IGF‐1 on disease risk, particularly in the brain (Ashpole *et al*., 2014), highlights a larger emerging paradox regarding the relevance of IGF‐1 to aging in humans.

Clearly, disruption of the insulin/IGF‐1 signaling pathway in lower organisms extends lifespan, while a decrease in growth, and hence body size in mammals, is also linked to longevity (Barzilai *et al*., 2012; Brown‐Borg & Bartke, 2012). However, while low peripheral IGF‐1 is linked to less cancer (Renehan *et al*., 2004), it is associated with increased risk for other age‐related diseases in humans (Sonntag *et al*., 2012). These observations, coupled with recent evidence that *daf2* mutants spend a proportionately greater time in a frail state, despite their longevity phenotype (Bansal *et al*., 2015), further highlight the complexity of this axis. Given the pleiotropic effects of IGF‐1 in the brain and periphery, many of which are beneficial, we propose that modulating this axis to promote healthspan in humans should be viewed as a ‘balance’ between its central and peripheral actions, to maximize its benefits, particularly in the CNS, while minimizing cancer risk in the periphery.

In older humans, increasing IGF‐1 levels in the brain is one approach toward optimizing this ‘balance’, which could be achieved via intranasal delivery. In rodents, intranasal IGF‐1 has been shown to rapidly and effectively gain entry to the CNS via the olfactory and trigeminal system (Thorne *et al*., 2004), and protect against Huntington disease and toxin‐induced brain injury (Cai *et al*., 2011; Lopes *et al*., 2014). Interestingly, intranasal insulin, which is under investigation in humans to treat metabolic (Heni *et al*., 2014; Gancheva *et al*., 2015) and cognitive decline (Claxton *et al*., 2015), has shown promise in healthy volunteers, but efficacy appears to wane in obese (Heni *et al*., 2014) and T2DM (Gancheva *et al*., 2015) patients, which may be the direct result of acquired central insulin resistance in these subjects. Thus, it seems plausible that increasing IGF‐1 in the brain may represent an important therapeutic alternative to circumvent central insulin resistance for the treatment of these diseases in older humans, a possibility that should be considered in future clinical trials. In summary, given the multifaceted benefits of IGF‐1 in the brain, we propose that strategies aimed at ‘tipping this balance’ centrally represent an ideal approach to modulate this axis for healthy aging and longevity in humans.

## Experimental procedures

### Animals

Young (3 month) and old (20 month) male SD rats were obtained from Harlan Laboratories (Madison, WI, USA). Animals were individually housed and allowed to acclimate for at least 1 week upon arrival before undergoing any procedures. All rats were housed at standard temperature (~ 22 °C) and humidity‐controlled conditions under a 14‐h L:10‐h D photoperiod and provided *ad libitum* access to water and chow. All experiments were approved by the Institutional Animal Care and Use Committee at the Albert Einstein College of Medicine.

### Phenotyping

Body weight was determined on a digital scale, and body composition was assessed by quantitative magnetic resonance for the determination of fat and lean mass in young and old animals (*n *= 8 per group) similar to the method previously described (Echo Medical Systems, Houston, TX, USA) (Huffman *et al*., 2008, 2013). Energy expenditure and substrate utilization were determined by indirect calorimetry as described (Huffman *et al*., 2007), based on O_2_ consumption and CO_2_ production, using a Rat Oxymax System (Columbus Instruments, Columbus, OH, USA). In brief, young and old animals (*n *= 8 per group) were placed into individual cages and allowed to acclimate for at least 72 h prior to the experiment. Data were then collected over two consecutive 24‐h periods and averaged. Animals were kept at their standard temperature and photoperiod and provided food and water *ad libitum* throughout the indirect calorimetry studies, and food intake was determined by the disappearance of food from the cage over a 48‐h period and averaged.

### Surgeries

In preparation for clamp studies, rats were sedated with 2% isoflurane for stereotactic placement of a steel‐guide cannula (Plastics One, Roanoke, VA, USA) reaching the 3rd ventricle (coordinates from bregma: +0.2 mm A/P, −9.0 mm D/V, 0.0 directly on the midsagittal suture) and the implant was secured in place with dental cement as described (Muzumdar *et al*., 2006a,b, 2009). Approximately 14 days later, rats were sedated for surgical placement of indwelling catheters into the right internal jugular vein and the left carotid artery as described (Muzumdar *et al*., 2006a,b; Einstein *et al*., 2008; Muzumdar *et al*., 2009; Einstein *et al*., 2010). Animals were treated with analgesic as needed postoperatively and recovery was monitored until animals were within 3–5% of their preoperative weight (~ 7 days) before conducting clamp studies.

### Hyperinsulinemic‐euglycemic clamp studies

We performed hyperinsulinemic‐euglycemic clamp studies with ICV infusion of aCSF, IGF‐1, or insulin, as described (Obici *et al*., 2002; Muzumdar *et al*., 2006a). Experiment 1 utilized only young animals treated with aCSF (*n *= 5), IGF‐1 (*n *= 6), and insulin (*n *= 9). Experiment 2 involving young and aged animals was performed in 5–7 animals per group. All studies were 360 min in duration and consisted of a 120‐min equilibration period, a 120‐min basal period, and a 120‐min hyperinsulinemic clamp period. At *t* = 0 min, animals were provided either a primed‐continuous ICV infusion of aCSF, 1 μg clinical‐grade human IGF‐I [0.3 μg bolus over 7.5 min, then 0.7 μg over 6 h (0.12 μg h^−1^)], or 30 μU regular porcine insulin (Sigma Aldrich, St. Louis, MO, USA) [7.5 μU bolus over 7.5 min, then 22.5 μU over 6 h (3.8 μU h^−1^)]. At *t* = 120 min, a primed‐continuous infusion of [3‐^3^H]‐glucose (20 μCi bolus, 0.2 μCi min^−1^ maintenance; NEN Life Science Products, Boston, MA, USA) was given into the jugular vein and maintained throughout the remainder of the study. At *t* = 240 min, a primed‐continuous intravenous infusion of regular insulin (3 mU kg^−1^ min^−1^) and somatostatin (1.5 g kg^−1^ min^−1^) was initiated, and a 25% glucose solution was given as needed to clamp the plasma glucose concentration at ~ 140–145 mg dL^−1^.

### Calculations of whole‐body glucose fluxes

Estimation of glucose fluxes during the basal and clamp periods were carried out as described previously (Muzumdar *et al*., 2006a; Einstein *et al*., 2008; Muzumdar *et al*., 2009; Einstein *et al*., 2010). Briefly, [3‐^3^H]‐glucose radioactivity was measured in duplicates in the supernatants of Ba(OH)_2_ and ZnSO_4_ precipitates of 50‐µL plasma samples after evaporation to dryness to eliminate tritiated water. Under steady‐state conditions of plasma glucose concentrations, the rate of glucose disappearance (*R*_d_) equals the rate of glucose appearance (*R*_a_). The latter was calculated as the ratio of the rate of infusion of [3‐^3^H]‐glucose (disintegrations per minute per minute) and the steady‐state serum [3‐^3^H]‐glucose SA (disintegrations per minute per milligram). The rate of glucose production was calculated as the difference between *R*_a_ and the GIR. The rates of glycolysis were estimated as described previously. Glycogen synthesis was estimated by subtracting the rate of glycolysis from the *R*_d_.

### Assays and analytical procedures

Plasma measures for metabolites and hormones were determined in young and old animals (*n *= 12 per group), following a 4‐ to 6‐h fast, as described (Muzumdar *et al*., 2006a, 2009; Einstein *et al*., 2010). In brief, plasma glucose concentration was determined under basal conditions and throughout the clamp with an Analox GM7 analyzer (Analox Inst., USA Inc., Lunenberg, MA, USA). Free fatty acids were determined using a colorimetric assay kit (Wako Diagnostics, Richmond, VA, USA). Endogenous insulin was measured by a rat/mouse ELISA (EMD Millipore, Inc., Billerica, MA, USA) with rat insulin standards, and clamp insulin levels were measured by a human ELISA (ALPCO, Inc., Salem, NH, USA) with human insulin standards. Endogenous IGF‐1 and IGFBP‐3 levels in plasma were measured using a validated ‘in‐house’ ELISA at the USC Aging Biomarker Service Core (Muzumdar *et al*., 2006a).

### Western blotting

MBH wedges were freshly isolated as described (Su *et al*., 2012). Total MBH protein was extracted with a modified RIPA buffer, and protein content was determined using the BCA protein assay (Sigma, St. Louis, MO, USA) with BSA as a standard. Western blotting was performed similarly as described (Huffman *et al*., 2008; Muzumdar *et al*., 2010). In brief, 20 μg of total protein was loaded onto 4–15% Bis–Tris stain‐free gels and electrophoresed at 120 V constant for 90 min (*n *= 12 per group). Prior to transfer, gels were imaged on a Bio‐Rad Chemidoc MP Imaging System (Bio‐Rad, Hercules, CA, USA) to measure total protein load. Protein was then wet‐transferred for 1 h at 100 V constant onto PVDF membranes, and equal transfer was routinely determined by Ponceau S stain. Membranes were then blocked in 5% milk in TBST (0.1% Tween) for 1 h prior to overnight incubation at 4 °C with an appropriate primary antibody, all from Cell Signaling (Danvers, MA, USA), including total Erk (1:1000; #4695), total Akt (#4691; 1:1000), total IGF‐1R (#9750; 1:500), and total InsRβ (#3025; 1:500). Following a 1‐h incubation with secondary antibody, Clarity Western ECL Substrate (Bio‐Rad) was applied, bands were visualized using a Bio‐Rad Chemidoc MP to first pixel saturation, and densitometry was performed using image lab 4.1 (Bio‐Rad).

### Statistics

All data were analyzed by independent sample *t*‐tests or by one‐way anova and followed up with planned contrasts when appropriate using spss (SPSS Inc., Chicago, IL, USA). All values are presented as means ± SE. A *P *≤ 0.05 was considered statistically significant.

## Conflict of interest

None declared.

## Funding

This work has been supported by NIA R00AG037574 and Einstein Startup Funds to D.M.H.; NIH P01AG021654, P30AG038072, R37AG18381 and the Paul Glenn Foundation for Medical Research to N.B.; and 1R01GM090311, 1R01ES020812, 1P01AG034906 to P.C. This work was also supported by the Einstein‐Sinai Diabetes Research Center (P30DK20541). P.A. is supported by a T32 Training Grant (T32AG23475).
